# Serum microRNA expression signatures as novel noninvasive biomarkers for prediction and prognosis of muscle-invasive bladder cancer

**DOI:** 10.18632/oncotarget.9166

**Published:** 2016-05-04

**Authors:** Xiumei Jiang, Lutao Du, Weili Duan, Rui Wang, Keqiang Yan, Lili Wang, Juan Li, Guixi Zheng, Xin Zhang, Yongmei Yang, Chuanxin Wang

**Affiliations:** ^1^ Department of Clinical Laboratory, Qilu Hospital, Shandong University, Jinan, 250012, Shandong Province, China; ^2^ Department of Urology, Qilu Hospital, Shandong University, Jinan, 250012, Shandong Province, China

**Keywords:** muscle-invasive bladder cancer, microRNA, prediction, prognosis, serum

## Abstract

Noninvasive biomarkers for predicting the risk of muscle-invasive bladder cancer (MIBC) may expedite appropriate therapy and reduce morbidity and cost. Genome-wide miRNA analysis by Miseq sequencing followed by two phases of reverse transcription quantitative real-time PCR (RT-qPCR) assays were performed on serum from 207 MIBC patients, 285 nonmuscle-invasive bladder cancer (NMIBC) patients and 193 controls. A four-miRNA panel (miR-422a-3p, miR-486-3p, miR-103a-3p and miR-27a-3p) was developed for MIBC prediction with an area under the receiver operating characteristic curve (AUC) of 0.894 (95% CI, 0.846-0.931) for training set. Prospective evaluation of the miRNA panel revealed an AUC of 0.880 (95% CI, 0.834 to 0.917) in validation set, which was significantly higher than those of grade and urine cytology (both *p* < 0.05). Moreover, Kaplan-Meier analysis showed that MIBC patients with low miR-486-3p and miR-103a-3p levels had worse overall survival (*p* = 0.002 and *p* = 0.034, respectively). Cox analysis indicated miR-486-3p and miR-103a-3p were independently associated with overall survival of MIBC (*p* = 0.042 and *p* = 0.021, respectively). In conclusion, serum miRNA signatures might have considerable clinical values in predicting and providing prognostic information for MIBC.

## INTRODUCTION

Bladder cancer (BC) is one of the most prevalent malignancies worldwide, with approximately 74,000 new cases and 16,000 new deaths in 2015 in the United States [1]. At initial presentation, 75% of tumors do not invade the muscle wall, whereas 30% will recur with muscle-invasive disease during follow up [2]. The remaining 25% of tumors present with muscle-invasive disease which features a high mortality rate [3]. Currently, standard test used to measure the local stage of BC is transurethral resection (TUR) [4]. This method could provide guide on treatment of non-muscle-invasive BC (NMIBC) and staging information for muscle-invasive BC (MIBC). However, this approach is invasive, uncomfortable and costly. Although urine cytology is widely used as noninvasive test for diagnosis of BC, its value in discriminating MIBC from NMIBC is limited owing to low accuracy [5]. Therefore, novel and noninvasive biomarkers are urgently needed to improve current strategies for prediction of tumor stage.

MicroRNAs (miRNAs) are a subset of small non-coding RNAs (typically 19-25 nucleotides in length) that increase and/or decrease gene expression at the post-transcriptional level [6, 7]. Different miRNAs could function as tumor suppressors or oncogenes, and their expression has been associated with genesis and invasion of cancer [8–11]. Previous studies demonstrated that numerous miRNAs exist stably in human serum and have potential roles in diagnosis, staging and prediction of outcomes in various types of cancer [12–17]. Recently, we have identified a serum miRNA profile (miR-152, miR-148b-3p, miR-3187-3p, miR-15b-5p, miR-27a-3p and miR-30a-5p) for diagnosis of BC via Miseq sequencing followed by two phases of reverse transcription quantitative real-time PCR (RT-qPCR) assays [18]. However, unique serum miRNA signatures that could predict tumor stage of BC have not been identified.

In the present study, we designed an investigation following the similar strategy to test the hypothesis that stage-specific miRNAs exist in serum and can be useful in predicting MIBC with the hope that such findings may guide therapeutic choice. Moreover, correlation between serum miRNAs and prognosis of MIBC was further assessed.

## RESULTS

### High-throughput sequencing of serum miRNAs from MIBC patients, NMIBC patients and controls

Among the 567 serum miRNAs that were scanned by Miseq sequencing (≥ 1 copy), 234, 278 and 193 miRNAs were detectable (≥ 10 copies) in MIBC group, NMIBC group and control group, respectively. Expression of a miRNA was considered altered in MIBC only if at least 50 copies were detected by Miseq sequencing together with significant deregulations larger than two-fold change in MIBC vs. Normal, NMIBC vs. Normal, and MIBC vs. NMIBC comparisons. Based on these criteria, 23 miRNAs were selected as differentially expressed (Supplementary Table S1). miR-152, miR-148b-3p, miR-3187-3p, miR-15b-5p, miR-27a-3p and miR-30a-5p were also selected because they had been shown to have diagnostic value for BC in our previous study [18]. Thus, 29 miRNAs were selected as candidates for further testing via RT-qPCR.

### Evaluation of miRNA expression by RT-qPCR

**Figure 1:**
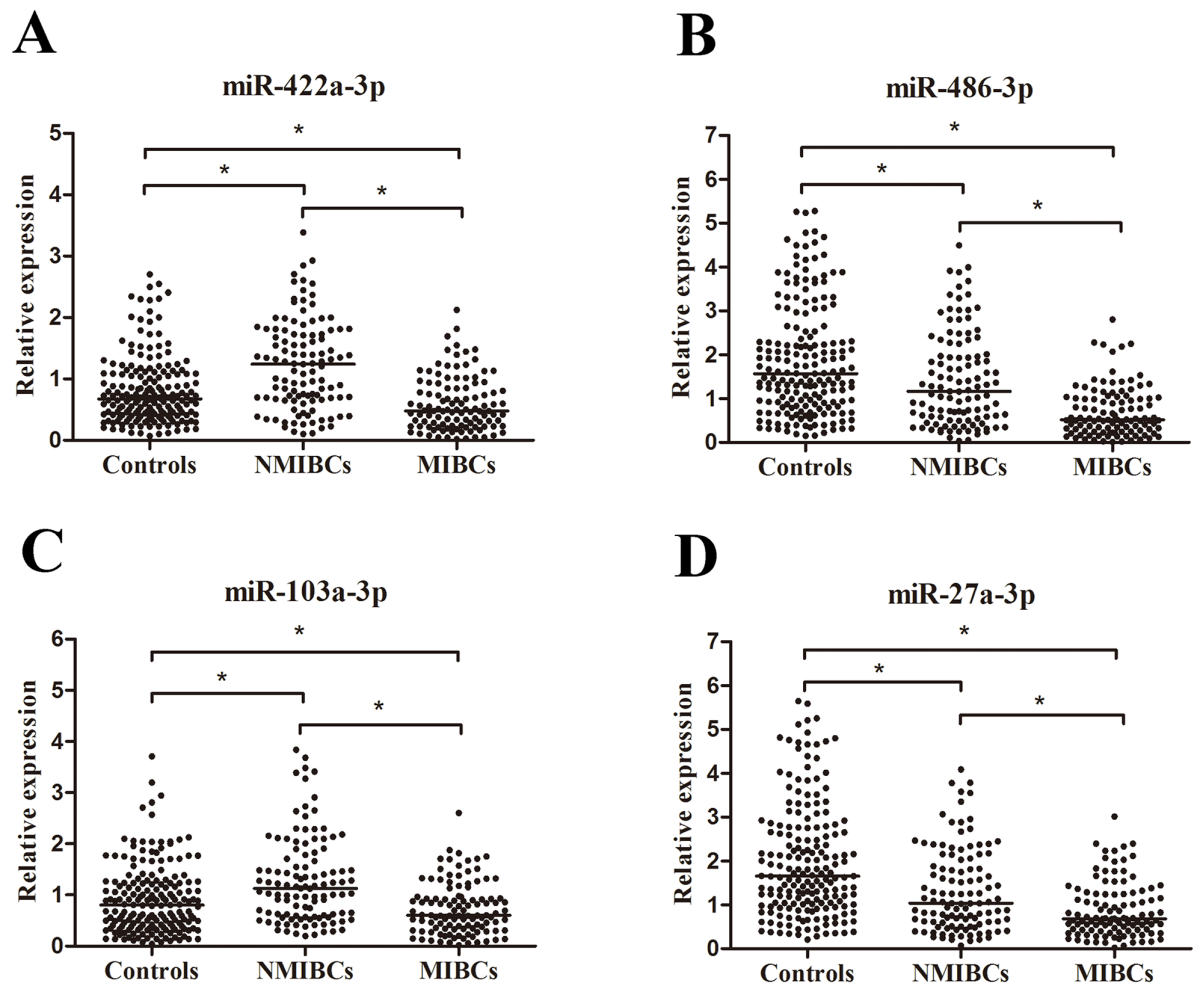
Concentrations of four miRNAs in MIBC patients (n = 111), NMIBC patients (n = 111) and control individuals (n = 187) using RT-qPCR assay in training set A-D. *p< 0.05

**Figure 2:**
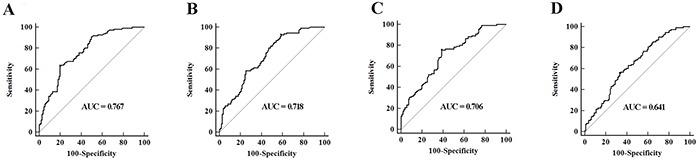
ROC curves analysis for detection of MIBC using A. miR-422a-3p, B. miR-486-3p, C. miR-103a-3p, D. miR-27a-3p in MIBC patients (n = 111), NMIBC patients (n = 111) and control individuals (n = 187) in training set

The 29 candidate miRNAs were first tested with RT-qPCR using an independent cohort of 40 MIBC patients, 40 NMIBC patients and 40 controls. Four miRNAs (miR-422a-3p, miR-486-3p, miR-103a-3p and miR-27a-3p) showed differential expression levels in MIBC vs. Normal, NMIBC vs. Normal, and MIBC vs. NMIBC comparisons (all at *p* < 0.05, Supplementary Figure S1). The expression profile of these four miRNAs was further evaluated by RT-qPCR on additional 71 MIBC patients, 71 NMIBC patients and 147 controls. The four miRNAs were significantly differently expression among MIBC, NMIBC and control groups (all at *p* < 0.05, Figure 1). The corresponding AUCs of these four miRNAs were 0.767, 0.718, 0.706, and 0.641, respectively (Figure 2).

The concentrations of the four miRNAs were further measured using the validation cohort consisted of 90 MIBC patients and 168 NMIBC patients. Alterations in miRNA expression pattern of validation set were consistent with those of training set (Table 1 and Supplementary Figure S2).

**Table 1:** Expression of four miRNAs in serum in NMIBC patients and MIBC patients in training set and validation set [median (interquartile range)]

miRNA	Training set			Validation set		
	NMIBCs	MIBCs	*p* value	NMIBCs	MIBCs	*p* value
miR-422a-3p	1.24 (0.67-1.79)	0.48 (0.23-0.85)	< 0.001	1.08 (0.65-1.53)	0.53 (0.33-0.79)	< 0.001
miR-486-3p	1.61 (0.54-2.01)	0.52 (0.25-1.01)	< 0.001	1.04 (0.70-1.74)	0.55 (0.36-0.92)	< 0.001
miR-103a-3p	1.27 (0.59-1.78)	0.60 (0.33-0.96)	< 0.001	0.97 (0.69-1.53)	0.65 (0.40-0.96)	< 0.001
miR-27a-3p	1.04 (0.59-2.03)	0.68 (0.42-1.25)	< 0.001	1.04 (0.67-1.81)	0.71 (0.47-1.23)	< 0.001

### Establishment of the predictive miRNA panel

**Figure 3:**
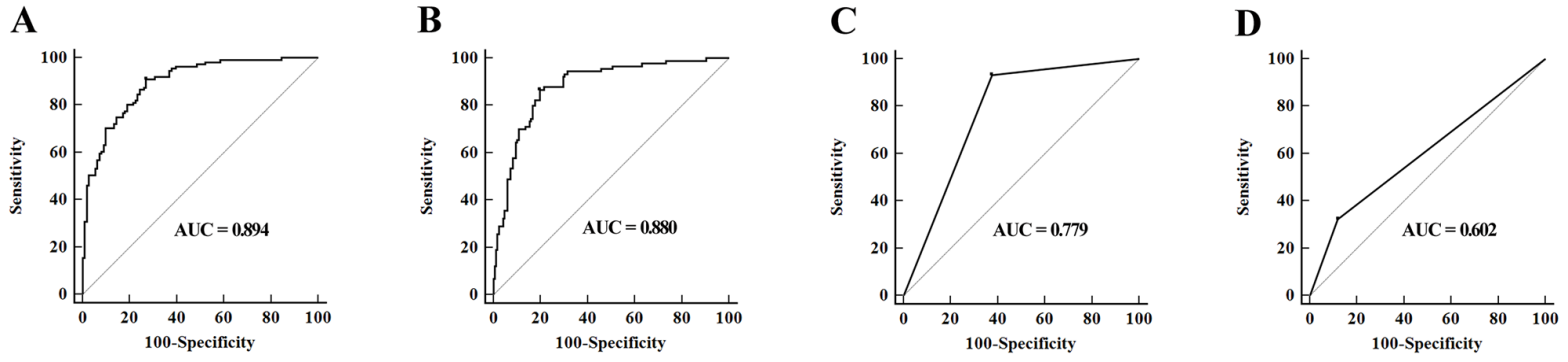
**A-B.** ROC curves for detection of MIBC using the four-miRNA panel in training set (A) and validation set (B); **C.** ROC curve analysis using grade for detection of MIBC in validation set; **D.** ROC curve analysis using urine cytology for prediction of MIBC in validation set.

A stepwise logistic regression model to estimate the risk of being predicted with MIBC was applied on training data set. The predicted probability of being diagnosed with MIBC from the logit model based on the four-miRNA panel, logit (*p* = MIBC) = − 1.5992 + (0.4938×miR-422a-3p) + (0.4197×miR-486-3p) + (0.4168×miR-103a-3p) + (0.2335×miR-27a-3p) was used to construct the ROC curve. The AUC of the four-miRNA panel was 0.894 (95% confidence interval [CI], 0.846 to 0.931) and the optimal cut-off value was 0.0282, providing a sensitivity of 90.99% and a specificity of 72.97% (Figure 3A). A threshold of 0.0282 was selected to ensure good predictive ability for MIBC.

### Validation of the predictive miRNA panel

The parameters estimated from the training set were used in a blind fashion to predict the probability of being diagnosed with MIBC for the independent validation data set. Using the classification threshold score of < 0.0282 derived above, 132 samples were identified as noninvasive and 126 samples were identified as invasive tumors. After unblinding 121 of the 168 noninvasive tumors [specificity, 70.06% (95%CI, 79.1 to 90.1)] and 79 of the 90 invasive tumors [sensitivity, 90.00% (95%CI, 81.9 to 95.3)] were correctly identified resulting in an AUC of 0.880 (95% CI, 0.834 to 0.917, Figure 3B). Analysis of these classification results demonstrated that accuracy for invasive tumors trended upwards the higher cancer stage (Table 2).

**Table 2:** Tumor staging classification performance of miRNA panel in BC patients in validation set

Tumor stage	Specificity (95% CI)	Sensitivity (95% CI)
Ta (n = 55)	61.82 (47.7-74.6)	—
T1 (n = 113)	71.68 (62.4-79.8)	—
T2 (n = 28)	—	78.57 (59.0-91.7)
T3 (n = 39)	—	87.18 (72.6-95.7)
T4 (n = 23)	—	95.65 (78.0-99.3)

Although low-grade BCs are rarely muscle-invasive, high-grade tumors can be either muscle-invasive or non-muscle-invasive. We thus evaluated tumor grade in classifying muscle-invasive from non-muscle-invasive tumors and ROC analysis revealed an AUC of 0.779 (95% CI, 0.724 to 0.828) (Figure 3C). The predictive performance of urine cytology in distinguishing MIBC from NMIBC was further evaluated on the validation set, and ROC analysis revealed an AUC of 0.602 (95% CI, 0.539 to 0.662) (Figure 3D). The AUC of the four-miRNA panel for MIBC was markedly higher than those of tumor grade and urine cytology (both *p* < 0.05).

### Correlations between miRNA expression levels and patient survival

**Figure 4:**
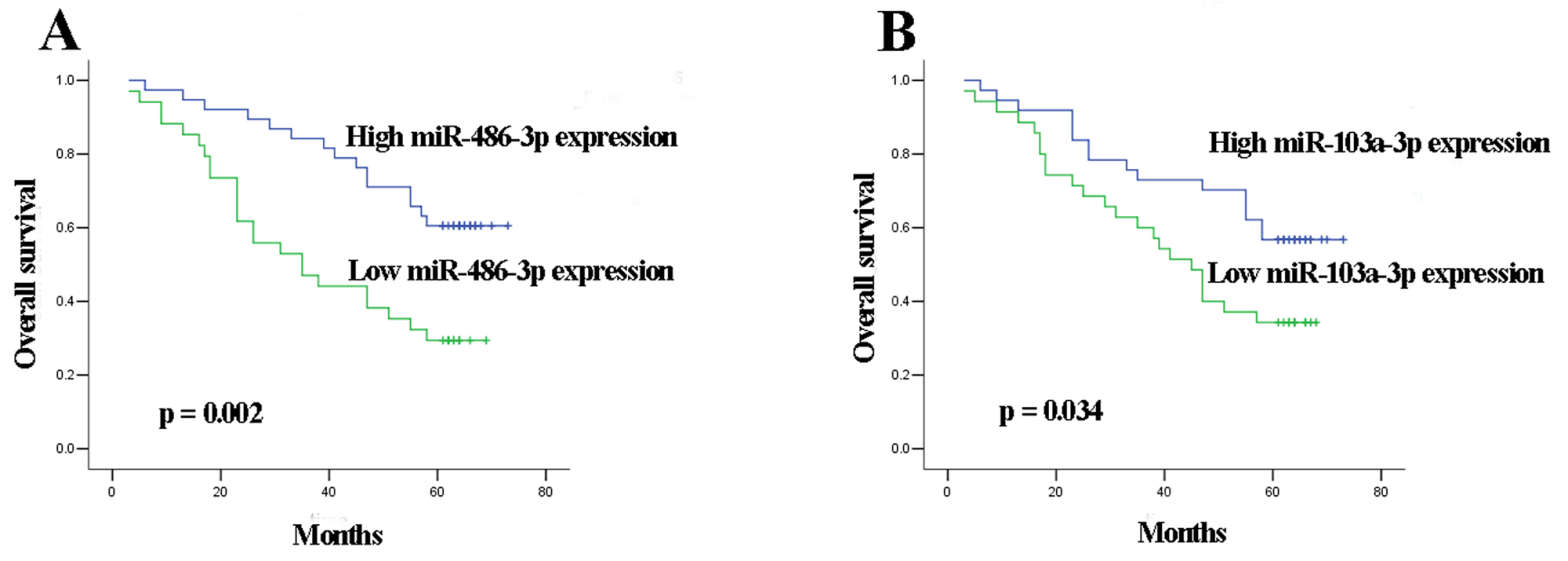
Kaplan-Meier curves for OS according to serum levels of A. miR-486-3p, B. miR-103a-3p in MIBC patients in validation set

In the validation phase, 18 of the 90 MIBC patients were lost to follow-up and survival analysis was performed on the remaining 72 MIBC patients. The median follow-up time was 56 (range 3-73) months. Kaplan-Meier survival analysis revealed that MIBC patients with low miR-486-3p and miR-103a-3p expression levels showed significantly reduced overall survival (OS) than those with high miR-486-3p and miR-103a-3p levels (*p* = 0.002 and *p* = 0.034, respectively) (Figure 4). Univariate Cox proportional hazards regression model analysis revealed that OS significantly correlated with miR-486-3p level (*p* = 0.004), miR-103a-3p level (*p* = 0.039), tumor stage (*p* = 0.005) and lymph node status (*p* = 0.031). Parameters significantly related to OS in the univariate analysis were put into the multivariate analysis to identify independent factors for prognoses. The results showed that miR-486-3p level (*p* = 0.042), miR-103a-3p level (*p* = 0.021), tumor stage (*p* = 0.030) and lymph node status (*p* = 0.025) maintained their significance as independent prognostic factors for OS of MIBC (Table 3).

**Table 3:** Univariate and multivariate Cox proportional hazards regression model analysis of OS in MIBC patients in validation set

Parameters	Categories	Univariate analysis		Multivariate analysis	
		HR (95% CI)	*p* value	HR (95% CI)	*p* value
Age	< 65 vs. ≥ 65	1.505 (0.789-2.872)	0.215		
Sex	Male vs. female	0.670 (0.262-1.715)	0.404		
Tumor stage	T2 vs. T3 vs. T4	1.830 (1.201-2.788)	0.005	1.682 (1.053-2.687)	0.030
Tumor grade	Low vs. high	1.002 (0.419-2.392)	0.997		
Lymph node metastasis	Negative vs. positive	2.285 (1.079-4.839)	0.031	2.418 (1.116-5.237)	0.025
miR-422a-3p expression	Low vs. high	1.083 (0.578-2.030)	0.804		
miR-486-3p expression	Low vs. high	0.380 (0.199-0.728)	0.004	0.500 (0.257-0.974)	0.042
miR-103a-3p expression	Low vs. high	0.509 (0.268-0.967)	0.039	0.460 (0.237-0.891)	0.021
miR-27a-3p expression	Low vs. high	1.660 (0.881-3.130)	0.117		

## DISCUSSION

In BC, identification of noninvasive and invasive phenotypes is vital to rational clinical management [19]. Previously, Schiffer et al. identified a panel of urinary peptides that seemed promising in predicting MIBC [5]. Yet, little was known about noninvasive miRNA biomarkers that can effectively accomplish this task. Catto et al. demonstrated that distinct miRNA alterations were associated with tumor progression of BC [20]. Wiklund et al. revealed coordinated epigenetic repression of the miR-200 family and miR-205 in invasive BC and suggested significant correlation between miR-200c silencing and T1 tumor progression [21]. In addition, miR-10a-5p has been shown to be down-regulated in tissues of Ta representing cases with stage progression [22]. Although these studies provide interesting insights into tumor pathology of BC, their effect in predicting tumor stage using serum still remains unclear. In the present study, our analysis revealed that miR-422a-3p, miR-486-3p, miR-103a-3p and miR-27a-3p were differently expressed in a MIBC-specific manner. Moreover, the four-miRNA signature demonstrated high accuracy for prediction of MIBC compared with tumor grade and traditional urine cytology. To our knowledge, this is the first study to characterize a serum miRNA expression signature for distinguishing muscle-invasive and non-muscle-invasive tumors of bladder by use of the genome-wide Miseq sequencing platform.

From a clinical perspective, the ability to predict muscle-invasive disease noninvasively could have important applications in urologic practice. Such biomarkers could offer pre-TUR information that may be useful for determination of the extent of TUR when MIBC is predicted. Under such conditions, when patients choose to have radical cystectomy, considering the benefit of neoadjuvant chemotherapy [23], such treatment can be implemented immediately. If patients want to undergo radiochemotherapy, complete TUR would be prepared. Furthermore, pathologists may have difficulty determining whether tumor has invaded the musclaris propria, which is crucial in choosing therapy. In such cases, these patients have to undergo a resection of the base of the tumor [24]. Here, a panel of serum miRNAs could provide relatively definite answer as to tumor stage evaluation, thus enabling a defined treatment pathway. Although numerous circulating miRNAs for detection of BC have been revealed [25–29], we provided the first evidence to support the hypothesis that serum miRNAs offer predictive value in distinguishing MIBC from NMIBC. Nevertheless, little was known about expression of serum miRNAs in recurrent MIBC and their predictive potential. As all patients with MIBC in this study were initially diagnosed, further studies are needed to validate whether the predictive miRNA panel could perform efficiently in recurrent cases. Therefore, our research should be deemed as an important first step but not the definitive answer for clinical utility of these putative biomarkers.

Among four miRNAs revealed in this study, some are involved in genesis and development of cancer. miR-486 has been shown to participant in general tumorigenesis by targeting the anti-apoptotic OLFM4 [30], SIRT1 [31], PIM-1 [32] and protumorigenic ARHGAP5 [33] in various types of cancer. Salah et al. proposed the miR-27a/miR-27a*pair as a potential diagnostic and therapeutic target in managing osteosarcoma metastases [34]. In addition, MTA1 knockdown could significantly inhabit the expression of miR-103, which might prohibit the invasive phenotype of lung cancer [35]. At tissue level, miR-103-1 has been previously identified as up-regulated in BC, indicating its potential involvement in the development and progression of BC [36]. Expression levels of miR-422a-3p, miR-486-3p and miR-27a-3p, however, have not been reported in tissues of BC. More researches on target genes of circulating miRNAs and complex mechanism that regulates the biogenesis of these four miRNAs in BC will be needed.

Currently, staging systems based on pathological grade and TNM stage are insufficient to predict clinical outcome. Different outcome for individuals with same tumor grade and stage calls for novel prognostic biomarkers and potential therapeutic targets [37, 38]. Several studies demonstrated that miRNA expression significantly correlated with tumor aggressiveness and patient survival in MIBC tissues [39, 40]. However, prognostic values of serum miRNAs for MIBC have not been explored. In the present study, low miR-486-3p and miR-103a-3p expression levels respectively correlated with shorter OS of MIBC. Taking a step further, Cox proportional hazards regression model analysis revealed that serum miR-486-3p and miR-103a-3p level were independent factors influencing OS of MIBC. Several investigators have reported similar findings in other types of cancer. Expression of miR-486 was found to be associated with cancer-specific mortality rate in non-small cell lung cancer [41]. Guo et al. demonstrated that miR-103/107 could serve as prognostic markers for OS of esophageal cancer [42]. Thus, we speculate that pretreatment serum levels of miR-486-3p and miR-103a-3p might also serve as new prognostic biomarkers for MIBC.

Some limitations should be acknowledged. First, origin of circulating miRNAs was not fully understood. Some investigators suggested serum miRNA profiles was not simply a default product of circulating blood cells but might derive from tissues affected by diseases such as cancer [43]. More focus on release mechanisms of miRNAs in tumorigenesis and progression of BC may be a valuable avenue for increasing diagnostic specificity. Moreover, it is still unknown whether the miRNA panel is capable of discriminating MIBC from other types of invasive tumors. In addition, our study has limited generalizability. Thus, confirmation of our findings in a multicenter trial of larger independent samples is the objective of ongoing work.

In conclusion, we defined a distinctive serum miRNA signature for prediction of MIBC and identified independent prognostic variables for MIBC. These findings may provide a foundation for development of a novel noninvasive test to predict MIBC and determination of innovative therapeutic strategies.

## MATERIALS AND METHODS

### Study design

**Figure 5:**
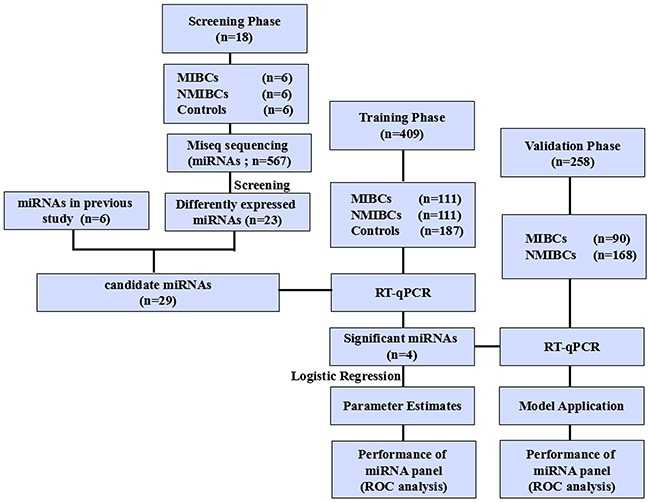
Study outline

A multiphase and case-control study was designed to identify serum miRNAs as surrogate biomarkers for discriminating MIBC from NMIBC (Figure 5). In the discovery phase, serum from 6 MIBC patients, 6 NMIBC patients and 6 healthy donors were respectively subjected to Miseq sequencing and miRNAs with significant differences in expression levels among three groups were identified. In the training phase, candidate miRNAs were first tested by RT-qPCR in an independent cohort of serum samples from 40 MIBC patients, 40 NMIBC patients and 40 controls. Subsequently, miRNAs differentially expressed among the three groups were further tested in 71 MIBC patients, 71 NMIBC patients and 147 controls. These 111 MIBC patients and 111 NMIBC patients were used to construct the predictive miRNAs panel based on logistic regression model for MIBC. In the validation phase, serum from another independent cohort of 90 MIBC patients and 168 NMIBC patients were prospectively entered into the discriminatory model to validate the predictive accuracy of the constructed algorithm. Meanwhile, urine samples were obtained for traditional urine cytology. Additionally, MIBC patients were followed up at intervals of 3 months during the first 2 years and 6 months up to the fifth year, and the date of the latest record retrieved was March 31, 2015. This study was approved by the Clinical Research Ethics Committee of Qilu Hospital, Shandong University and informed consent was obtained from each participant.

### Patients and control subjects

Patients with BC were recruited in the Department of Urology of Qilu Hospital, Shandong University between January 2005 and February 2010. Samples from BC patients were collected at the day before radical cystectomy and/or TUR. Tumor stage was determined according to the 2002 UICC TNM classification of BC and tumor grade was defined according to the WHO 2004 grading scheme. All MIBC patients were initially diagnosed with muscle-invasive diseases. Control participants without history of BC were recruited from a large pool of individuals seeking a routine health checkup at the Healthy Physical Examination Centre of Qilu Hospital, Shandong University. People who showed no evidence of disease were selected as tumor-free controls. The demographic and clinical features of participants are summarized in Supplementary Table S2.

### Serum preparation

Venous blood samples were collected by vena puncture from each participant before any treatment. Serum was separated via centrifugation at 1,600 g for 10 min at 4°C within 2 hr of collection, followed by a second centrifugation at 16,000 g for 10 min at 4°C to eliminate residual cells debris. Supernatant serum was then stored at −80°C till use.

### Miseq sequencing

For Miseq sequencing, total RNA was extracted and purified using miRNeasy Mini Kit (Qiagen, Valencia, CA, USA) following the protocol provided by the manufacturer. Briefly, a pair of adaptors was ligated sequentially to the 3′ and 5′ ends of miRNA, and the ligated miRNA molecules were amplified by RT-qPCR to construct a cDNA library. Quality of the library was measured by the KAPA RT-qPCR kit and cDNAs with concentrations of higher than 1 nM and no dimmer contamination were used directly for sequencing analysis (Miseq sequencer, Illumina, San Diego, CA, USA). The final reads of miRNA were identified by normalization with the total reads of all called miRNAs in the sample. Bioinformatics analysis was conducted by searching against the miRBase 17.0 to determine known mature miRNAs.

### Quantification of miRNAs by RT-qPCR analysis

The 2× preparation buffer that consisted of 2.5% Tween 20 (EMD Chemicals, Gibbstown, NJ), 50 mmol/L Tris (Sigma-Aldrich, St.Louis, MO), and 1mmol/L EDTA (Sigma-Aldrich, St.Louis, MO) was prepared [44]. First, 3 μl of serum were mixed with 3μl of 2× preparation buffer. Then, the 6μl mixture were reverse transcribed in a 20-μl reverse transcription (RT) reaction volume system including 10μl of 2× miRNA Reaction Buffer Mix, 2μl of miRNA Primescript RT Enzyme Mix and 2μl of 0.1% BSA. The reactions were incubated at 37°C for 60min, followed by 85°C for 5s and 4°C for 60min. The generated cDNAs were centrifuged at 16,000 g for 10min at 4°C. The 25-μl RT-qPCR reaction system contained 12.5μl of SYBR Premix Ex Taq II, 0.5μl of Dye II, 2μl of 5μM of forward primer, 1μl of 10μM of Uni-miR RT-qPCR Primer, 7μl of ddH_2_O and 2μl of template cDNA. The reactions were incubated at 95°C for 30s, followed by 45 cycles of 95°C for 5s and 57°C for 34s. All reactions were performed in triplicate in ABI PRISM 7500 Sequence Detection System (Applied Biosystems, Foster City, CA) using the SYBR PrimeScript miRNA RT-qPCR Kit (Takara Bio Inc). Specificity of the RT-qPCR product was confirmed with melting curve analysis and Ct values were determined using default threshold setting. miRNAs with a Ct value of more than 35 and a detection rate of less than 75% in any group were excluded from further analyses. The combination of miR-16-5p and miR-193a-5p were used as reference genes [45]. The relative expression fold change was calculated by using the 2^−ΔΔCt^ method [46].

### Urine cytology determination

Midstream urine sediments were separated via centrifugation at 1,300 g for 10 min and immediately processed for cytological examinations by two cytopathologists in a blinded fashion. The cytology was categorized as positive if cancer cells or cells with atypical changes indicating malignancy were found, and negative if mild to moderate atypical changes were observed.

### Statistical analysis

Kolmogorov-Smirnov test was used to determine the normality of the distribution of data in each group. Data were presented as median (interquartile range). Difference of miRNA levels among multiple groups in Miseq sequencing was determined by Bonferroni adjustment method. Mann-Whitney U tests were employed to compare differences of miRNAs between two groups in further validation. Receiver operating characteristic (ROC) curves were established to discriminate MIBC from NMIBC. Area under the ROC curve (AUC) was used as an accuracy index for evaluating the predictive performance of constructed miRNA panel [47]. The Youden index (sensitivity + specificity − 1) was used to determine the optimal cutoff point [48]. Survival curves were estimated with Kaplan-Meier method and comparisons were conducted using log-rank test. Cox proportional hazards regression model was employed to identify independent prognostic factors. Matlab software (Matlab, R2013a) was employed for logistic regression analysis, MedCalc 9.3.9.0 (MedCalc, Mariakerke, Belgium) was used for ROC analysis, and other analysis was performed by SPSS version 17.0 software (SPSS Inc., Chicago, IL). Statistical significance was defined as two-sided *p* value < 0.05.

## SUPPLEMENTARY FIGURES AND TABLES


