# Supplementary material for: Serum microRNA expression signatures as novel noninvasive biomarkers for prediction and prognosis of muscle-invasive bladder cancer
Source: Oncotarget. 2016 May 4;7(24):36733–42. doi: 10.18632/oncotarget.9166 (PMC5095035; doi:10.18632/oncotarget.9166)
Supplement: Supplementary file 1 [file oncotarget-07-36733-s001.pdf]

SUPPLEMENTARY FIGURES AND TABLES

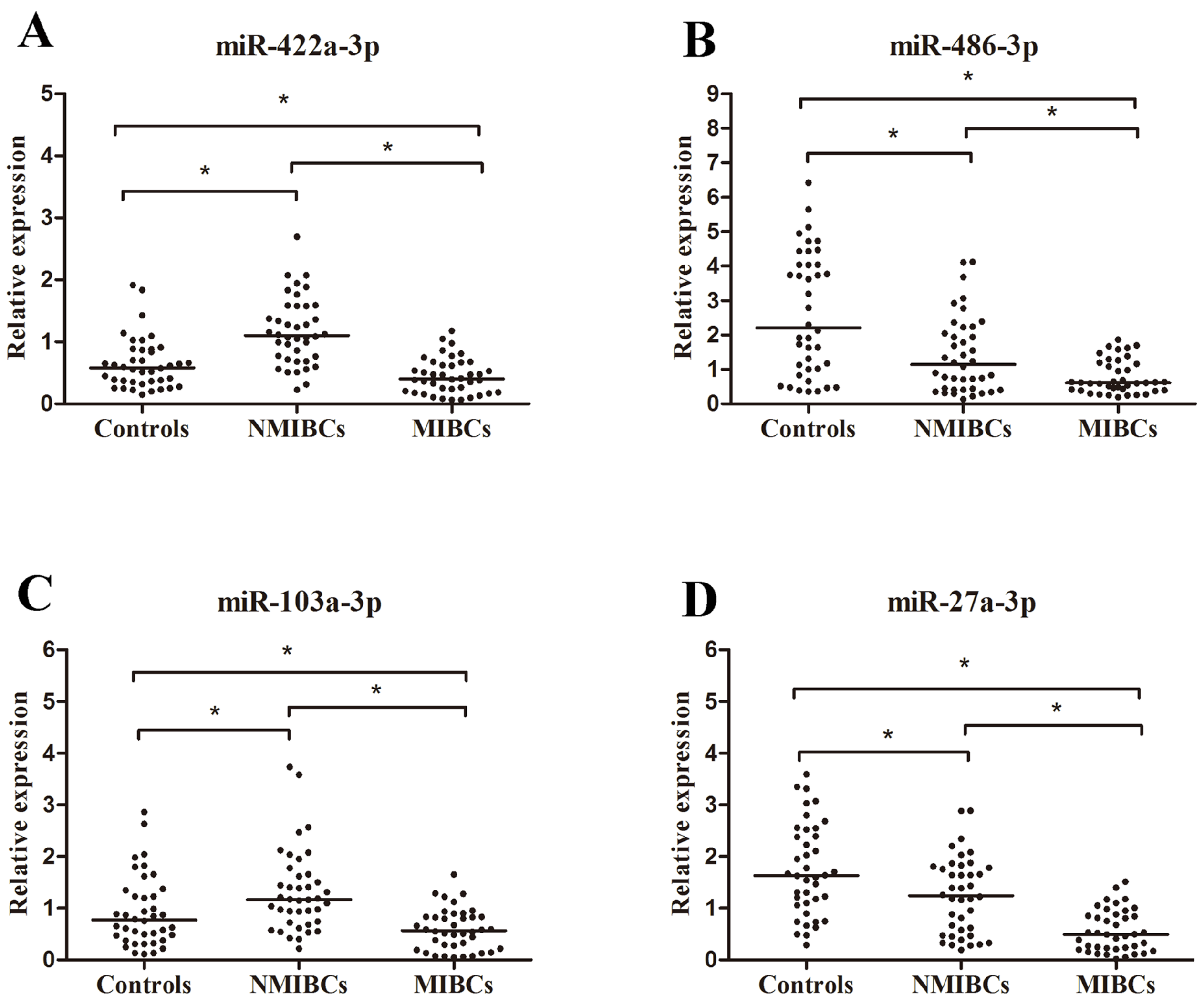

Supplementary Figure S1: Concentrations of four selected serum miRNAs in MIBC patients (n = 40), NMIBC patients (n = 40) and control individuals (n = 40) using RT-qPCR assay in training set A-D. $*p < 0.05$.

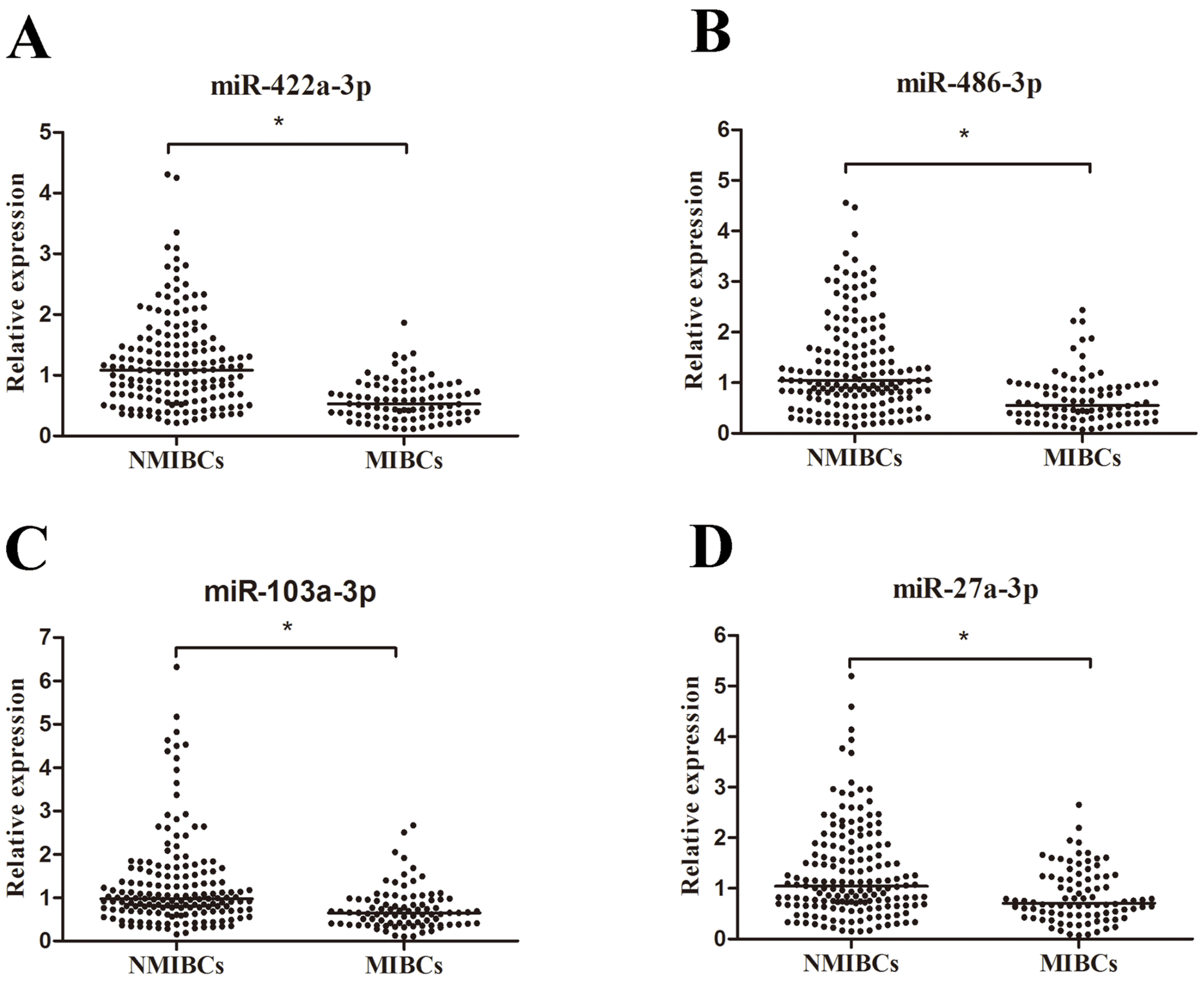

Supplementary Figure S2: Concentrations of four selected serum miRNAs in MIBC patients (n = 90) and NMIBC patients (n = 168) using RT-qPCR assay in validation set A-D. *$p < 0.05$.

Supplementary Table S1: Candidate altered miRNAs in serum of MIBC group, NMIBC group and control group determined by Miseq sequencing

Dysregulated miRNAs	Fold change (MIBC vs Control)	*p* value (MIBC vs Control)	Fold change (NMIBC vs Control)	*p* value (NMIBC vs Control)	Fold change (MIBC vs NMIBC)	*p* value (MIBC vs NMIBC)
miR-125b-5p	3.06	< 0.05	6.65	< 0.05	0.46	< 0.05
miR-130a-3p	5.78	< 0.05	2.13	< 0.05	2.71	< 0.05
miR-4732-3p	0.33	< 0.05	2.53	< 0.05	0.13	< 0.05
miR-3065-3p	7.98	< 0.05	2.14	< 0.05	3.73	< 0.05
miR-422a-3p	0.38	< 0.05	3.95	< 0.05	0.10	< 0.05
miR-378a-3p	0.22	< 0.05	0.48	< 0.05	0.46	< 0.05
miR-122-5p	0.47	< 0.05	0.14	< 0.05	3.36	< 0.05
miR-136-5p	8.45	< 0.05	2.02	< 0.05	4.18	< 0.05
miR-22-3p	2.88	< 0.05	10.27	< 0.05	0.28	< 0.05
miR-146a-5p	0.16	< 0.05	0.39	< 0.05	0.41	< 0.05
miR-205-5p	0.31	< 0.05	0.12	< 0.05	2.58	< 0.05
miR-769-5p	0.35	< 0.05	2.92	< 0.05	0.12	< 0.05
miR-19a-3p	2.14	< 0.05	4.86	< 0.05	0.44	< 0.05
miR-107	0.12	< 0.05	0.37	< 0.05	0.32	< 0.05
miR-320a	0.24	< 0.05	0.49	< 0.05	0.49	< 0.05
miR-423-5p	0.13	< 0.05	0.38	< 0.05	0.34	< 0.05
miR-486-3p	0.12	< 0.05	0.43	< 0.05	0.28	< 0.05
miR-574-3p	6.98	< 0.05	3.05	< 0.05	2.29	< 0.05
miR-4429	2.73	< 0.05	6.21	< 0.05	0.44	< 0.05
miR-205-5p	0.46	< 0.05	2.13	< 0.05	0.22	< 0.05
miR-103a-3p	0.26	< 0.05	2.91	< 0.05	0.09	< 0.05
miR-23a-3p	0.34	< 0.05	0.15	< 0.05	2.27	< 0.05
miR-362-5p	6.97	< 0.05	2.11	< 0.05	3.30	< 0.05

Supplementary Table S2: Characteristics of study participants in training set and validation set [median (interquartile range)]

Variable	Training set (n=409)	Validation set (n=258)
**Control (number)**	187	—
**Age (years)**	64 (56-73)	—
**Sex**		—
Male	149 (79.68%)	—
Female	38 (20.32%)	—
**BC (number)**	222	258
**Age (years)**	66 (58-79)	65 (59-77)
**Sex**		
Male	182 (81.98%)	205 (79.46%)
Female	40 (18.02%)	53 (20.54%)
**Tumor stage**		
Ta	43 (19.37%)	55 (21.32%)
T1	68 (30.63%)	113 (43.80%)
T2	31 (13.96%)	28 (10.85%)
T3	50 (22.52%)	39 (15.12%)
T4	30 (13.51%)	23 (8.91%)
**Tumor grade**		
Low grade	101 (45.50%)	110 (42.64%)
High grade	121 (54.50%)	148 (57.36%)
**Lymph node metastasis**		
Negative	190 (85.59%)	228 (88.37%)
Positive	32 (14.41%)	30 (11.63%)
